# Translation, cultural adaptation and assessment of psychometrics properties of the Extended Version of the Nordic Musculoskeletal Questionnaire (NMQ-E) in Persian language speaking people

**DOI:** 10.1186/s12891-024-07192-9

**Published:** 2024-01-23

**Authors:** Hamid Reza Mokhtarinia, Zahra Sadat Javadi Hosseini, Seyed Mohammadreza Shokouhyan, Charles Philip Gabel

**Affiliations:** 1 Department of Ergonomics, University of Social Welfare and Rehabilitation Sciences, Tehran, Iran; 2 Centre Hospitalier Universitaire Vaudois (CHUV), Department of Musculoskeletal Medicine (DAL), Swiss BioMotion Lab, University of Lausanne (UNIL), Lausanne, Switzerland; 3 Access Physiotherapy, Coolum Beach, Sunshine Coast, QLD Australia

**Keywords:** Musculoskeletal Pain, Cross-cultural adaptation, Validation, Physiotherapy, Persian

## Abstract

**Background:**

To translate and cross-culturally adapt the Extended Version of the Nordic Musculoskeletal Questionnaire (NMQ-E) into Persian (NMQ-E-P) and evaluate the psychometric properties in a general population with different occupational tasks across nine body regions.

**Methods:**

This cross-sectional study was designed according to the standard guidelines and the COSMIN checklist. The NMQ-E-P was achieved through forward and backward translation methods and consensus to produce the final draft. A Persian-speaking population (*n* = 571, age 38.24 ± 7.65 years, female = 46.2%) was recruited from industries and office workers with three occupational task inclusion criteria: assembly, office, and lifting. Psychometric properties included validity for face (from confirmed clarity, simplicity, and readability), content (via the content validity index); and construct (through known group validity); additionally, the properties of internal consistency (Cronbach’s α); and test-retest reliability (Kappa coefficient of agreement) were considered.

**Results:**

No significant issues during the translation process were found. The NMQ-E-P showed adequate internal consistency for all regions (α ≥ 0.87). The test-retest reliability was examined with Kappa agreement correlation coefficient and all items, except ankle regions, showed very good agreements (Kappa coefficient = 0.87-1.0). Excellent ICC values were obtained for quantitative variables (ICC > 0.88) and good construct validity was revealed (*p* < 0.001).

**Conclusion:**

The Persian version of the NMQ-E has very good validity and reliability and can be used by researchers and professionals to evaluate the prevalence of MSDs in nine body regions simultaneously.

## Background

Work-related musculoskeletal disorders (WMSDs) include diseases that are characterized by pain, ache, disability, and impairment in soft tissues. These tissues include muscles, nerves, bones, tendons, and cartilage, and impairment may be caused or aggravated by work [1]. In general, it is reported that musculoskeletal disorders account for 20-30% of work absenteeism days in different countries, which is a relatively high and concerning statistic. Heavy burdens are placed on societies because of these work absenteeism costs that are a consequence of such WMDs [2, 3]. In Iran, a prevalence rate range of 38.1-50% for the upper and lower back regions is reported respectively [4].

Due to the multifactorial etiology of MSDs such as physical, psychosocial, personal and organizational [5, 6], several body regions are usually exposed to the injury simultaneously. Several body sites and work-related site‐specific disorders such as the back, neck, shoulders, hands, wrists, knee, hips and nonspecific neck and low back pain, osteoarthritis, epicondylitis, nerve entrapment, plantar fasciitis are reported in the workplace [7–9]. Because of the limitation of the previous instruments to gather greater data regarding the prevalence of musculoskeletal pain in various regions of the body, the need for new reliable and valid instruments with the ability to simultaneously generate data from multi-body regions is evident. In contrast, WMSDs should be differentiated and screened appropriately from other disorders caused by diseases such as fibromyalgia or arthritis [10] or by falls, slips, trips, or similar incidents [11].

There are a variety of instruments to screen or predict MSDs in general and occupational settings. Observational methods are the most common way for practitioners to predict and identify physical exposures within a workplace [12]. There are some limitations to observational methods which include low reliability [13], based on concepts of an external observer [14], solely for risk assessment usage, time-consuming [15], and the need for a combination of several observational methods to assess the risk [16].

Self-report “questionnaires” are the second method most commonly used by ergonomists to screen or predict MSDs. These are straightforward to use, suitable for a large number of participants, and are low in time demand, as well as being applicable to a wide range of tasks at a low cost [17]. One of the most common and widely used tools in this regard is the general Standardized Nordic Musculoskeletal Questionnaire (NMQ). The NMQ was presented by Kuorinka et al. and used extensively to quantify musculoskeletal pain and related activity limitations [18]. However, though the NMQ was initially introduced over 20 years ago, its psychometric properties have been updated and evaluated over recent years [19]. Some psychometric properties, such as reliability and validity, were evaluated in different languages such as *Portuguese* [20], *Italian* [21], *Greek* [22] *Turkish* [23], *and Iranian* [24]. The original questionnaire was developed for occupational settings but recent psychometrics evaluations were performed on specific groups such as the patients or workers rather than general populations or mixed populations [25].

Dawson et al. provided a new version of the NMQ in English, the extended NMQ (NMQ-E) that is applicable for nine body regions and able to extract data about the prevalence, severity and consequences of MSDs on the daily life of the individual [19]. The NMQ-E has the ability to measure the point, annual, and lifetime prevalence of musculoskeletal symptoms [19]. It is a one-page user-friendly questionnaire that is completed in a short time for these nine body regions. To measure the same parameters by a specific outcome measure in different cultures and languages, cross-cultural adaptation must be performed [26]. Although there is a need for such a tool that can simultaneously evaluate several body areas, the translation and cultural adaptation to the Persian language have yet to be performed. In contrast, several studies [27–29] have already used the NMQ-E version in Persian, but the psychometric evaluation of the Persian NMQ-E version has not been completed using a rigorous scientific methodology. Therefore, this study aimed to translate and cross-culturally adapt the NMQ-E for use in Persian, and to determine the psychometric properties of the Persian translated version (NMQ-E-P) which included evaluation of reliability (test-retest reliability and internal consistency) and validity (face, content, construct and convergent validity) in a general population with different occupational tasks.

## Methods

### Study design

We conducted the methods and results analysis of this cross-sectional study according to the standard published papers for outcome measurement instruments for the workplace setting [30, 31] and the COSMIN (COnsensus-based Standards for the selection of health status Measurement INstruments) criteria [32]. The consent from the developer of the original NMQ-E, Ana Dawson was obtained. The ethical committee of the University of Social Welfare and Rehabilitation Sciences approved the study (IR.USWR.REC.1401.260). Informed written consent was obtained from all participants. A 2-step approach was used in this study that included: (1) translation and cross-cultural adaptation, and (2) psychometrics evaluation within a general population group with different occupational tasks.

### Participants

The participants were recruited between 2022 and 2023 from different industries and offices to select occupations that included lifting tasks, office working tasks, and montage assembling tasks. According to the published guidelines [33], a subject to item ratio method is used for sample size determination in psychometric validation studies. It was shown that around 92% of the articles reported a subject to item ratio of ≥ 2, whereas 25% had a ratio of ≥ 20 [34]. We selected a ratio of 5 per item with a 15% dropout as our ratio precedent to ensure a minimum sample size. Consequently, with *n* = 571 (99 items of NMQ-E ) [18, 35] we exceeded the required minimum of *n* = 495. A final sample of convenience (*n* = 571) was used and participants were placed into one of the three groups: lifting (*n* = 120), office workers (*n* = 260) and montage (*n* = 211). The general mean age was 38.24 ± 7.65, and BMI of 24.47 ± 2.90, with a female 46.2%.

The eligible criteria were: (1) aged 20–55 years; (2) employed in the same job for the preceding 12 months; (3) willingness to participate in the study; and (4) ability to read, write and understand the questions in the Persian language.

### Instruments

#### The Extended Version of the Nordic Musculoskeletal Questionnaire (NMQ-E)

The E-NMQ was developed originally by Anna P. Dawson et al. The NMQ-E [18] consists of 99 questions in a dichotomous yes/no response option that provides information on the onset, prevalence and consequences of musculoskeletal pain in nine separate body regions (the neck, shoulder, upper back, elbow, wrist/hand, low back, hip/thigh, knee, ankle/foot) [35]. The questions are respectively ordered about the respondents’ lifetime, prevalence, and consequences of pain. Questions for a body region should be responded to horizontally, before progressing to the next body region in the provision of the new raw data. The questionnaire is completed in a short time requiring approximately 10 to 15 min [25].

#### National Aeronautics and Space Administration Task load index (NASA-TLX)

The NASA-TLX is a multi-dimensional self-report scale that is designed to estimate the mental workload in occupational settings. Six subscales of physical demand, mental demand, temporal demand, effort, performance, and frustration are incorporated [36]. Each dimension is rated for the level of demand on a 7-point scale. Increments of high, medium, and low estimates for each point result in 21 graduations on the scales. To discriminate the mental workload between the participants, the total workload score was compared between the three subgroups. Further, the physical subscale was used as an effective and straightforward approach to detect the physical workload in the three different groups as a consequence of the high correlations reported between the weighted overall score and the Raw-TLX indices [37, 38]. The NASA-TLX is used extensively in Persian studies [39] and its Persian version was used in the current study [40]. The two NASA_TLX subscales, physical and mental, were used to screen the level of load in a simple way to confirm the difference between the three groups.

#### Translation and cross-cultural adaptation of the NMQ-E

Translation and cultural adaption processes were conducted according to the accepted guidelines in five steps including forward translation, synthesis, backward translation, consolidation, and pre-final step testing [41]. Two independent native bilingual Persian translators performed the forward translation. One translator (T1) had a PhD in physiotherapy with experience in outcome measures translation and the second translator was blinded to the research circumstances (T2). A brief explanation of the tool’s use, target population, and the purpose of the translation was provided to the translators to increase the final product quality. The first translator, with the help of one of the researchers, synthesized a general version of the NMQ-E. Two additional bilingual translators, blinded to the original version, back-translated the synthesized version into English (T3, T4). An expert committee composed of an occupational therapist, a physiotherapist, two ergonomists, an occupational medicine specialist, four translators, and a methodologist reviewed all translations, the consensus version, and the original questionnaire. Following the discussion on the semantic, conceptual equivalence and idiomatic discrepancies, consensus on a pre-final NMQ-E was obtained. The pre-final version was sent to the developer of the questionnaire (A. Dawson) to confirm its equivalency with the original version.

In the pilot stage, to determine the understandability, simplicity, clarity and readability of the questionnaire by its user, face validity was conducted as a subjective measure through the interview process [42]. Fifteen participants from the same working environment were instructed to read and complete the questionnaire under the supervision of one of the researchers. Any difficulty understanding or ambiguity of phrases or words was requested to be highlighted. Participants were also asked for possible suggestions and alternatives for ambiguous items. The consensus on the clarity of language, simplicity, and readability was obtained through a focus group session and the final Persian version of the NMQ-E was created for the process of psychometric evaluation.

#### Psychometric assessment of the NMQ-E

##### Face validity

Through a qualitative analysis of the comments that were provided by the 15 subjects in the pilot study, the face validity of the Persian NMQ-E was obtained.

##### Content validity

Content validity depends on the extent to which an empirical measure reflects a specific domain of content. Initially, the Content Validity Index at item level (I-CVI) was determined through the proportion of expert agreement on the individual items. The average of the I-CVIs was calculated and considered as the average scale-level-CVI (S-CVI/Ave) [43]. To confirm the content validity eight experts from different disciplines including a physiotherapist, ergonomist, occupational therapist and orthopedic surgeon, evaluated and rated the content relevancy on a 4-point Likert scale (1 = not relevant, 4 = very relevant). The proportion of experts that gave a rating of 3–4 was considered as I-CVIs and S-CVI [44, 45]. An acceptable cut-off value for the eight experts is reported about 78% [44].

##### Construct validity

Construct validity was tested verifying two hypotheses. The first is that the physical workload is different between the three occupational subgroups (known group validity). Hence, the participants with higher physical workload reported a greater number of body regions being involved during the previous month or year. The second hypothesis was the existence of a correlation between the number of involved body regions during the preceding month with the number of visits to doctors and the amount of medication taken by the individual.

##### Reliability

Two concepts of test-retest reliability and internal consistency were considered for reliability evaluation. The same questionnaire was completed twice by 110 participants (mean age 28.40 ± 7.32 years) who were selected randomly from the sample of 571 participants. Repeated measures were made at an interval of 1–3 days during which the participant’s condition remained stable. The NMQ-E completion was performed within the individual’s workplace at the same time and same place. Further, Cohen’s unweighted Kappa was used for dichotomous variables within the test-retest reliability. Kappa values were categorized as poor (0 to 0.2), fair (0.21 to 0.40), moderate (0.41 to 0.60), good (0.61 to 0.80), and very good (0.81 to 1.00) [46]. The intraclass correlation coefficient (ICC_2,1_) was used to determine the test-retest reliability of continuous variables. An ICC_2,1_ value > 0.70 indicated excellent reliability [47].

The internal consistency of each item was assessed with Cronbach’s Alpha (α) and the related 95% confidence intervals. An α value ≥ 0.70 is considered acceptable and indicates satisfactory internal consistency reliability (0.7–0.8: acceptable, 0.8–0.9: good > 0.9: excellent) [45, 47].

All statistical analyses were calculated using the statistical package for Social Science version 22 (SPSS 22) for Windows. Statistical significance was set at *P* < 0.05.

## Results

### Phase-I: translation and cross-cultural adaptation

There were no significant issues while translating the NMQ-E into Persian. The chiropractic discipline as an academic course is not available to the Iranian general population and consequently, they are not familiar with it, hence it was removed from the questionnaire. During the face validity, the participants generally stated that the instructions of the questionnaire were simple to understand and complete. Participants could complete the tool quickly without difficulty. Therefore, the final version in Persian was obtained without significant changes to the original version.

### Participants

Six hundred subjects were invited to participate in the study voluntarily. The information data of 29 participants was not complete and they were excluded from the study. Among the remaining 571 participants, the rate of acceptability was calculated by the proportion of missing item responses, with high overall completion rates (≥ 90%) taken as evidence of questionnaire acceptability. The average rate of completion for all items was above 94% indicating the acceptability of the questionnaire. The mean ± SD of age and Body Mass Index (BMI) of the participants were 38.24 ± 7.65 years and 24.47 ± 2.90 kg/m2, respectively. A total of *n* = 381 (53.8%) of respondents were male. The demographic characteristics of the participants (*n* = 571), in general, and according to three different occupational tasks are presented in Table 1. The characters of categorical variables are presented as frequency (n) and percentage (%), while the continuous variables as mean and standard deviation (SD).

**Table 1:** Demographic descriptive characters of general and three group participants

Variable		Montage Tasks (n = 211)	Office Tasks (n = 260)	Lifting Tasks (n = 120)	Total (n = 571)
Age (year)		34.04 ± 6.73	42.54 ± 6.50	36.30 ± 6.52	38.24 ± 7.65
Height (m)		169.22 ± 9.77	166.06 ± 8.22	174/83 ± 7.05	168.97 ± 9.19
Weight (kg)		68.73 ± 10.80	68.58 ± 10.18	75.15 ± 9.21	69.97 ± 10.53
BMI		23.98 ± 3.07	24.81 ± 2.76	24.59 ± 2.80	24.47 ± 2.90
Daily working time(h)		44.64 ± 1.85	42.27 ± 13.12	43.81 ± 2.40	43.43 ± 2.79
Total working time (yr)		13.92 ± 4.16	16.24 ± 6.88	8.35 ± 6.41	10.24 ± 8.13
Child number		0.92 ± 1.09	1.08 ± 0.88	1.30 ± 0.93	1.07 ± 0.98
Gender	Male	124 (58.8)	74 (28.5)	120(100)	318 (53.8)
	Female	87 (41.2)	186(71.5)	0	273 (46.2)
Marital status	Married Single	109 (51.7) 102 (48.3)	190 (73.1) 70 (26.9	102 (85) 18 (15)	401 (67.9 190 (32.1)
Education	Under Diploma Associate and bachelor’s Degree Post graduated	151 (71.6) 60 (28.4) 0	12 (4.60) 109 (41.9) 139 (53.5)	106 (88.30) 14 (11.70) 0	270 (45.70) 182 (30.80) 139 (32.10)
Cigarette smoking	Yes No	23 (10.90) 188 (89.10)	3 (1.20) 257 (98.8	41 (34.20) 79 (65.80)	67 (11.30) 524 (88.70)
Regularly exercise	Yes No	37 (17.50) 174 (82.50)	40 (15.40) 220 (84.60)	54 (45) 66 (55)	130 (22.2) 461 (77.80)
Medical history	Yes No	29 (13.70) 182 (86.30)	39 (15) 221 (85)	2 (1.70) 118 (98.30)	70 (11.80) 521 (88.20)
Surgery history	Yes No	71 (33.60) 140 (66.40)	24 (9.20) 236 (90.80)	2 (1.70) 118 (98.30)	97 (16.40) 494 (83.60)

The most common body region with the highest lifetime prevalence of MSDs was the low back (*n* = 671, 58.7%) for the general population. Further, the lifetime prevalence in three different groups of office workers, montage workers and lifting workers were reported as 68.8%, 44.1%, and 62.5%, respectively. The body area reported with the most pain during the preceding year and month was related to the low back with 48.7%, and 48.9%, respectively. In the point prevalence category, the neck was the highest prevalent pain-experiencing region (27.9%). The results of MSDs prevalence for general and separated occupational tasks are provided in Tables 2 and 3, respectively.

**Table 2:** Prevalence of MSDs In General participants in percentage (%)

Regions	Have you ever had trouble (ache, pain or discomfort)	Have you ever been hospitalized because of the trouble?	Have you ever had to change jobs or duties because of the trouble?	Have you had trouble (ache, pain or discomfort) at any time during the last 12 months?	Have you had trouble (ache, pain or discomfort) at any time during the last months (4 weeks)?	Have you had trouble (ache, pain or discomfort) today?	During the last 12 months have you at any time:			
							Been prevented from doing your normal work (at home or away from home) because of trouble?	Seen a doctor, physiotherapist or other such person because of trouble?	Taken medication because of trouble?	Taken sick leave from work/studies because of trouble?
Neck	48.1	0.5	6.1	38.9	37.4	27.9	6.3	13.5	14.14	12.4
Shoulders	38.4	0.8	3.7	31.1	31.1	22.8	4.7	9.0	11.2	8.8
Upper Back	36.4	0.8	3.6	28.9	28.6	20.3	4.1	7.6	9.3	9.0
Elbows	12.2	0.3	0.5	9.5	9.6	6.3	2.2	2.7	3.6	2.7
Wrist/hands	38.2	2.0	5.4	30.3	30.8	23.5	6.1	11.2	12.0	10.0
Low back	58.7	3.6	6.1	48.7	48.9	27.1	12.5	26.2	25.0	22.2
Hips/thighs	19.0	1.2	1.5	14.4	15.1	11.0	3.4	4.7	6.1	6.3
Knees	43.1	1.4	2.5	33.0	36.2	25.7	4.9	12.5	14.4	10.3
Ankles/feet	32.0	1.0	3.7	24.5	36.6	21.0	3.2	8.3	8.5	9.1

**Table 3:** Prevalence of MSDs In three groups of participants in percentage (%)

Regions		Have you ever had trouble (ache, pain or discomfort)	Have you ever been hospitalized because of the trouble?	Have you ever had to change jobs or duties because of the trouble?	Have you had trouble (ache, pain or discomfort) at any time during the last 12 months?	Have you had trouble (ache, pain or discomfort) at any time during the last months (4 weeks)?	Have you had trouble (ache, pain or discomfort) today?	During the last 12 months have you at any time:			
								Been prevented from doing your normal work (at home or away from home) because of trouble?	Seen a doctor, physiotherapist or other such person because of trouble?	Taken medication because of trouble?	Taken sick leave from work/studies because of trouble?
Neck	Office workers	59.2	1.2	5	43.8	40.8	20.0	8.8	18.8	16.9	5.0
	Manual Montague	44.5	0	6.2	39.8	39.3	38.4	1.4	4.3	9	23.2
	Lifting workers	30.0	0	8.3	26.7	26.7	26.7	9.2	18.3	18.3	9.2
Shoulders	Office workers	52.3	0.8	4.2	37.3	37.7	20.8	5.8	13.5	13.5	3.8
	Manual Montague	36.6	0.9	4.3	32.7	32.7	30.3	1.4	2.8	7.1	16.6
	Lifting workers	15.0	0.8	1.7	15	14.2	14.2	8.3	10.0	13.3	5.8
Upper Back	Office workers	52.3	1.9	4.2	37.3	37.3	18.8	4.6	10.4	10.8	3.8
	Manual Montague	25.6	0	2.4	23.7	23.7	23.2	0.5	1.4	5.2	15.6
	Lifting workers	20.8	0	4.2	20.0	18.3	18.3	9.2	12.5	13.3	8.3
Elbows	Office workers	15.4	0.8	0.4	9.6	9.2	3.8	0.8	1.9	2.7	1.2
	Manual Montague	6.2	0	0.9	6.6	6.6	4.7	0	0	1.4	4.3
	Lifting workers	15.8	0	0	14.2	15.8	14.2	9.2	9.2	9.2	3.3
Wrist/hands	Office workers	46.2	3.8	5.8	30.0	31.2	16.5	6.9	16.5	19.2	4.6
	Manual Montague	30.8	0.9	5.2	28.4	28.4	27.0	0.5	2.4	2.8	16.6
	Lifting workers	34.2	0	5	34.2	34.2	32.5	14.2	19.2	25.0	11.7
Low back	Office workers	68.8	6.2	8.1	50.4	51.2	26.5	13.5	33.8	29.6	17.3
	Manual Montague	44.1	0.9	5.7	41.2	40.8	39.3	1.4	8.5	14.2	28.9
	Lifting workers	62.5	2.5	2.5	58.3	58.3	55.8	30.0	40.8	34.2	20.8
Hips/thighs	Office workers	25.8	2.3	2.7	16.9	18.1	9.6	5.0	7.7	9.2	3.8
	Manual Montague	14.2	0.5	0.9	12.8	13.3	12.3	0.5	0.9	2.8	10.9
	Lifting workers	12.5	0	0	11.7	11.7	11.7	5.0	5.0	5.0	3.3
Knees	Office workers	58.5	3.1	4.2	37.7	44.6	22.3	1.9	16.5	19.2	4.6
	Manual Montague	31.8	0	1.4	30.3	30.8	28.9	0.9	3.3	5.2	19.9
	Lifting workers	30.0	0	0.8	27.5	27.5	27.5	7.5	20.0	20.0	5.8
Ankles/feet	Office workers	38.1	2.3	3.5	22.3	27.3	15.8	4.2	11.5	12.3	3.1
	Manual Montague	30.8	0	4.7	29.4	28.9	28.0	0.5	1.4	2.8	19.4
	Lifting workers	20.8	0	2.5	20.8	20.8	20.0	5.8	13.3	10.0	4.2

### Reliability

The results of the ICC_2.1_ values for the age of onset of the ‘trouble’ question, showed excellent test-retest reliability (ICC > 0.88) with the lowest values being reported for the upper back (0.88) and the highest values for the wrist and hips (0.98). The Kappa agreement correlation coefficient for the remaining questions, including the prevalence questions and severity questions, are presented in Tables 4 and 5. For all body regions, except the ankle region, very good agreements were obtained. For some variables, it was not possible to compute the agreement correlation coefficient test, because the same and constant answers were given in two conditions. The internal consistency of all regions exceeded 0.70 indicating an acceptable satisfactory internal consistency reliability as available in Table 4.

**Table 4:** Test-retest reliability of NMQ-E in an combined occupational settings (*n* = 110), Age of Onset and Prevalence Questions and internal consistency

Regions	Age at the onset of trouble			Life time prevalence	Annual Prevalence	Month prevalence	Point prevalence	Internal consistency	
	ICC(2,1) (95% CI))	SEM (yrs)	MDC_95_	Kappa Coefficient	Kappa Coefficient	Kappa Coefficient	Kappa Coefficient	α Cronbach	
Neck	0.93 (0.87–0.98)	1.65	4.57	1.00	1.00	0.96	0.94	0.95	
Shoulders	0.92 (0.81–0.99)	0.98	2.71	0.94	0.98	0.93	0.98	0.87	
Upper Back	0.88 (0.80–0.93)	1.25	3.46	1.00	0.98	0.95	1.00	0.87	
Elbows	0.96 (0.93-1.00)	0.78	2.16	0.98	1.00	0.96	0.97	0.88	
Wrist/hands	0.98 (0.95–0.99)	1.35	3.73	0.95	0.95	1.00	1.00	0.88	
Low back	0.94 (0.92–0.99)	0.89	2.46	0.93	0.92	0.94	1.00	0.92	
Hips/thighs	0.98 (0.89-1.00)	1.25	3.46	0.91	0.98	0.94	0.99	0.92	
Knees	0.96 (0.74–0.99)	1.88	5.20	1.00	1.00	0.90	1.00	0.91	
Ankles/feet	0.91 (0.88–0.99)	1.29	3.57	0.89	0.96	0.94	0.98	0.87	

**Table 5:** The Kappa coefficient and Confidence Interval values indicating test-retest reliability of NMQ-E in a combined occupational settings (*n* = 110): Questions about Consequences of Pain

Regions	Lifetime hospitalization	Lifetime Changed Jobs	Annual Prevention of Normal work	Annual Visit by Health Profession	Annual Medication	Annual Sick Leave
Neck	1.00	1.00	0.89 (0.72–1.06)	0.95 (0.88–1.02)	0.94 (0.85–1.03)	*
Shoulders	1.00	1.00	1.00	0.89 (0.73–1.05)	0.95 (0.88–1.02)	0.97 (0.92–1.02)
Upper Back	*	*	0.98 (0.97–0.99)	1.00	1.00	0.95 (0.86–1.03)
Elbows	1.00	1.00	1.00	0.96 (0.89–1.03)	1.00	0.98 (0.95–1.01)
Wrist/hands	*	1.00	0.98 (0.95–1.01)	1.00	1.00	*
Low back	1.00	0.93 (0.84–1.02)	1.00	1.00	0.87 (0.66–1.08)	1.00
Hips/thighs	*	1.00	0.98 (0.88-1.01)	1.00	1.00	*
Knees	0.84 (0.7–0.98)	0.80 (0.61–0.99)	1.00	0.87 (0.68–1.06)	0.92 (0.79–1.05)	0.87 (0.64–1.1)
Ankles/feet	0.78 (0.39–1.17)	0.73 (0.44–1.02)	1.00	0.68 (0.54–0.82)	0.78	*

* Cannot compute the Kappa coefficient

### Content validity

All eight experts endorsed the relevancy and validity of the NMQ-E-P items (I-CVI = 1.00; S-CVI-Ave = 1.00).

### Construct validity

A one-way ANOVA was performed to compare the effect of groups on the mental and physical demand variables. A one-way ANOVA revealed that there was a statistically significant difference in the physical workload variable between at least two groups (F(2, 566) = [172.9]p, *p* < 0.001). No significant difference was shown for the mental workload between groups (F(2, 566) = [4.05], *p* = 0.1). Tukey’s HSD Test for multiple comparisons found that the mean value of physical workload was significantly different between each paired group (Table 6).

**Table 6:** Multiple Comparisons Bonferroni test in three groups for dependent variables

Dependent Variable	Groups (I)	Groups (J)	Mean Difference (I-J)	*P* value	95% Confidence Interval	
					Lower Bound	Upper Bound
Physical Demand	Montage task	Office task	6.011^*^	< 0.001	4.94	7.08
		Lifting task	-3.512^*^	< 0.001	-4.92	-2.10
	Lifting task	Office task	9.523^*^	< 0.001	8.15	10.89
Number of involved regions annually	Montage task	office task	0.40361*	0.045	0.0087	0.7985
		Lifting task	− 0.14515	0.031	− 0.3448	0.6351
	Lifting task	office worker	0.54876*	0.023	0.0757	1.0219
Number of involved regions monthly	Montage task	office worker	0.52758*	0.010	0.1293	0.9259
		Lifting task	− 0.11839	0.049	− 0.3918	0.6286
	Lifting task	office task	0.64597*	0.031	0.0424	1.2496

*. The mean difference is significant at the 0.05 level

Our hypothesis was that, the number of body regions involved in the last year and month were greater in the groups with higher physical workload. A one-way ANOVA result showed a statistically significant difference in the number of involved body regions during the last year (F(2, 586) = [3.37], *p* = 0.035) and month (F(2, 575) = [4.91], *p* = 0.008) between at least two groups. Accordingly, Tukey’s HSD Test for multiple comparisons found that the mean value for the number of involved regions was significantly different between each paired group (Table 6).

The results of the second hypothesis analysis showed that there was a significant correlation between the number of involved body regions and the number of visits to the doctor (*r* = 0.52, CI = 0.45–0.60, *P* < 0.001) and the amount of medication taken (*r* = 0.46, CI_95_ = 0.39–0.54, *P* < 0.001) in general sample.

## Discussion

The purpose of the current study was to develop a screening tool for musculoskeletal symptoms and the assessment of severity in Persian language-speaking people. Further, the related psychometric properties, including reliability and validity, were appropriately evaluated using the COSMIN standards. The NMQ-E is extracted from the NMQ and, because of its user-friendly and comprehensive format, has been translated into different languages in different formats, both online and paper-based, for use in varied cultural and linguistic settings [25, 35, 48]. During the process of translation, no significant difficulties were found except for the chiropractic phrase that was removed as this specialization does not have an academic center in Iran and is not a familiar term for Iranian Persian-speaking people. This change and revision parallels that which was applied in the Turkish version for the same cultural reasons [25].

The minimum values of the Kappa Coefficient for lifetime, annual, month and day-time prevalence were respectively 0.89, 0.92, 0.93, and 0.94, which indicated very good questionnaire stability. It appears that ‘memory decay’, a recognized and normal phenomenon in daily life, is only a minor contributing factor to the results; this is demonstrated by the test-retest interval time being very short (1–3 days) during a period where the participants’ health status was unchanged. This 1–3 day test-retest interval was selected as the subjects’ health condition should not have changed. In contrast Dawson et al. [19] used a time interval of 24 h and Pugh used 4 to 7 days [35].

In comparison with previous research, our reliability results demonstrated relatively lower Kappa coefficient values in comparison with the original [19]. In contrast to both the original and the Hebrew version, in this study, ‘lifetime prevalence’ demonstrated the lowest whereas point prevalence displayed the highest values [19, 48]. This could be due to cultural variation of the difference in the retest period, factors that will need to be considered in future research.

The NMQ-E internal consistency was not examined by Dawson et al., whereas in this study, the alpha range of 0.87–0.95 was obtained, which highlighted the relationship between items without the presence of item-redundancy. In line with our study, alpha values > 0.78 and ICC values > 0.88 were also reported in the Turkish version [25]. This is in contrast to the Hebrew version where lower internal consistency was reported [48], indicating no relationship was present between items. In a modified NMQ-E version in a nursing population, high internal consistency was reported for each region and the related subscales of Severity of symptoms and Impact on activities [35]. Generally, the values of internal consistency should be considered cautiously, and are probably consequences of chance, because there is no logical relationship in this setting for a consistency between knee pain and neck pain. The ICC value for the age of onset of the trouble item was very similar to the original version [19] and the modified NMQ-E [35]. It seems that the online NMQ-E version may have sufficient psychometric properties for health professional use.

In eight items that are consequences of pain, we cannot compute the Kappa coefficient because of the total negative responses, a finding that was also present in the original version [19].

To the best of our knowledge, there are only two other NMQ-E versions, the Turkish [25] and the online Hebrew [48]. Additionally, a modified NMQ-E online version was provided to measure nurses’ fitness [35]. In this study, the content and construct validity were respectively evaluated using CVI and hypothesis testing. With the current study findings, there is consistency with the Turkish [25] and modified NMQ-E [35] versions, while the content validity was evaluated and determined as adequate through the use of the CVI.

To evaluate the construct validity hypothesis testing was used, as there appears to be no available tool that can simultaneously examine several body regions in terms of the prevalence and severity of symptoms. In the Turkish version, the Cornell Musculoskeletal Discomfort Questionnaire (CMDQ) was used to assess construct validity and a correlation between the two questionnaires was noted and confirmed [25]. However, this CMDQ criterion and the comparative methodology were not selected in this study as the validity of such a comparison is questioned due to the binary response option (yes or no). As a consequence, a correlation analysis between the items is not statistically sound and should not be made. Further, all body regions evaluated in the NMQ-E are not equally represented or present in the CMDQ, so a direct comparison by pairs is not possible. A final limitation in using the CMDQ questionnaire is the lack of a standardized Persian version. Consequently, the evaluation of the construct validity was determined through hypothesis testing. One of our hypotheses was that the subjects with higher physical workload were more likely to have a greater number of involved body regions. This can be ascertained through the use of ANOVA statistical analysis which demonstrated that physical workload was significantly different among the three groups in the following order: lifting tasks > montage tasks > office work tasks. Accordingly, we confirmed our expectation related to the difference of involved body regions in the different groups. Further, we anticipated that patients with higher physical workloads, and consequently a higher number of involved body regions, would likely have a greater incidence of doctor visits and require more medicine. This was demonstrated through the positive correlation.

The NMQ-E is originally extracted from the NMQ to gather more data on the prevalence rate and impacts of musculoskeletal pain on daily activity. Therefore, the psychometrics properties of the NMQ are comparable to some sections of the NMQ-E. The NMQ has been translated into Chinese [49], Turkish [50], Persian [51], Brazilian Portuguese [52] and Greek [53]. The reported Kappa coefficients in these studies were mostly between 0.63 and 1.0 indicating good reliability, which is similar to our findings in the related sections.

This study was conducted to develop the NMQ-E-P and then assess the psychometric properties. In general, the reliability statistics are adequately strong and sufficient for use as a screening instrument in research and epidemiological studies of Persian subjects.

### **Limitation and strength**

Several limitations in this study are noted. The distribution of participants in each group was not equal due simply to the random sampling methodology. Another limitation was that no criterion or similar Persian NMQ-E instrument was available for criterion validity evaluation. An interval of 1–3 days for test-retest reliability evaluation was another limitation of the study. An interval of more than three-days will increase the chance of changing the subjects’ health condition. Further, the dichotomous nature of the NMQ-E items limits the factor analysis of the instrument as the responses of the items are independent, without a defined total score.

One of the study’s strengths was that the questionnaire was administered to a combined population with a relatively high prevalence of musculoskeletal disorders. Consequently, the generalizability of the results is higher than that for other language versions. Additionally, the evaluation of content and construct validity provides further support for this questionnaire and strengthens the current study.

## Conclusion

The Persian version of the NMQ-E was shown to have very good validity and reliability in a sample of Iranian Persian-speaking workers from both office and industry settings. The NMQ-E-P was shown to have sound characteristics and application for the industrial and office settings it is intended. The NMQ-E-P can be used by researchers and professionals to evaluate the prevalence of MSDs in nine body regions simultaneously. Further research is required in prospective populations to verify these psychometric findings and to determine the relevance of change and any other variables that can be altered over time.

## Data Availability

Data is available on request due to privacy/ethical restrictions. The data that support the findings of this study are available on request from the corresponding authors, HRM. The data are not publicly available due to information that could potentially compromise the privacy of research participants.

## Abbreviations

NMQ-E: Extended Version of the Nordic Musculoskeletal Questionnaire
NMQ-E-P: Persian Version of the Extended Nordic Musculoskeletal Questionnaire
ICC: intraclass correlation coefficient
WMSDs: Work-related musculoskeletal disorders
COSMIN: COnsensus-based Standards for the selection of health status Measurement Instruments
NASA-TLX: National Aeronautics and Space Administration Task Load Index
CVI: Content Validity Index
I-CVI: item CVI
S-CVI/Ave: average scale-level-CVI
α: Cronbach’s Alpha
BMI: Body Mass Index
SD: standard deviation
CMDQ: Cornell Musculoskeletal Discomfort Questionnaire

## References

[1] Aptel M, Aublet-Cuvelier A, Cnockaert JC. Work-related musculoskeletal disorders of the upper limb. Joint Bone Spine, 2002. 69(6): p. 546 – 55. [10.1016/s1297-319x(02)00450-5]. 10.1016/s1297-319x(02)00450-5 12537261

[2] Panush RS. Chap. 35 - occupational and recreational Musculoskeletal disorders. In: Firestein GS, et al. editors. Kelley and Firestein’s Textbook of Rheumatology (Tenth Edition). Elsevier; 2017. pp. 520–32.

[3] Luger T (2019). Work-break schedules for preventing musculoskeletal symptoms and disorders in healthy workers. Cochrane Database Syst Rev.

[4] Parno A (2017). The prevalence of occupational musculoskeletal disorders in Iran: a meta-analysis study. Work.

[5] Weale V (2022). Workplace musculoskeletal disorders: a systematic review and key stakeholder interviews on the use of comprehensive risk management approaches. Int J Ind Ergon.

[6] Descatha A et al. Occupational determinants of musculoskeletal disorders. Handbook of disability, work and health, 2020: p. 169–188.

[7] Neupane, S., C.-H. Nygård, and J. Oakman, Work-related determinants of multi-site musculoskeletal pain among employees in&nbsp;the health care sector. Work, 2016. 54: p. 689–697. [10.3233/WOR-162320]. 10.3233/WOR-162320 27315409

[8] Jacquier-Bret J, Gorce P (2023). Prevalence of body area work-related Musculoskeletal disorders among Healthcare professionals: a systematic review. Int J Environ Res Public Health.

[9] Mokhtarinia HR, Zareiyan A, Gabel CP (2021). Cross-cultural adaptation, validity, and reliability of the Persian version of the s functional index. Hand Therapy.

[10] Güler MA, Çakit MO (2020). Decreased chronic widespread Pain on Nonworking days might help Differentiate Work-Related Musculoskeletal disorders from Fibromyalgia: a cross-sectional study of working females. Arch Rheumatol.

[11] Chenna D et al. Prevalence of musculoskeletal disorders among dental healthcare providers: A systematic review and meta-analysis. F1000Res, 2022. 11: p. 1062. [PMC9709350] [10.12688/f1000research.124904.2]. 10.12688/f1000research.124904.1 PMC9709350 36505095

[12] Eliasson K, Lind CM, Nyman T (2019). Factors influencing ergonomists’ use of observation-based risk-assessment tools. Work.

[13] Eliasson K (2017). Inter- and intra- observer reliability of risk assessment of repetitive work without an explicit method. Appl Ergon.

[14] Andreas G-WJ, Johanssons E (2018). Observational methods for assessing ergonomic risks for work-related musculoskeletal disorders. A scoping review. Revista Ciencias De La Salud.

[15] Takala E-P et al. Systematic evaluation of observational methods assessing biomechanical exposures at work. Scand J Work Environ Health, 2010: p. 3–24. 10.5271/sjweh.2876 19953213

[16] Takala EP (2010). Systematic evaluation of observational methods assessing biomechanical exposures at work. Scand J Work Environ Health.

[17] G.C. Ergonomic methods for assessing exposure to risk factors for work-related musculoskeletal disorders. Occup Med. 2005;55(3):190–9. 10.1093/occmed/kqi082. 10.1093/occmed/kqi082 15857898

[18] Kuorinka I (1987). Standardised nordic questionnaires for the analysis of musculoskeletal symptoms. Appl Ergon.

[19] Anna P, Dawson EJS, y, Paul W. Hodges,* and Simon Stewart, Development and Test–Retest Reliability of an Extended Version of the Nordic Musculoskeletal Questionnaire (NMQ-E): A Screening Instrument for Musculoskeletal Pain. The Journal of Pain, 2009. 10(4): p. 517–526. 10.1016/j.jpain.2008.11.008 19345154

[20] Mesquita CC, Ribeiro JC, Moreira P (2010). Portuguese version of the standardized nordic musculoskeletal questionnaire: cross cultural and reliability. J Public Health.

[21] Gobba F (2008). Italian translation and validation of the nordic IRSST standardized questionnaire for the analysis of musculoskeletal symptoms. La Medicina Del Lavoro.

[22] Antonopoulou M (2004). Translation and standardisation into Greek of the standardised general nordic questionnaire for the musculoskeletal symptoms. Eur J Gen Pract.

[23] Kahraman T, Genç A, Göz E (2016). The nordic Musculoskeletal Questionnaire: cross-cultural adaptation into Turkish assessing its psychometric properties. Disabil Rehabil.

[24] Namnik N (2016). Validity and reliability of Persian version of the specific nordic questionnaire in Iranian industrial workers. Work.

[25] Alaca N (2019). Translation and cross-cultural adaptation of the extended version of the nordic musculoskeletal questionnaire into Turkish. J Musculoskel Neuronal Interact.

[26] Mokhtarinia HR (2018). Cross-cultural adaptation, validity, and reliability of the Persian version of the spine functional index. Health Qual Life Outcomes.

[27] Sharifi AS, Danesh MK, Gholamnia R (2022). Improvements in musculoskeletal symptoms, mental workload and mental fatigue: effects of a multicomponent ergonomic intervention among call center workers. Work.

[28] Akhavan Z, Kheirkhah A (2022). Reducing the ergonomic load on nurses using integrated job rotation and shift scheduling plan: a multi-objective optimization approach. Iran J Ergon.

[29] Mortazavi SS (2022). Relationship between Musculoskeletal Disorder and School Bag Characteristics among mentally retarded students. Pajouhan Sci J.

[30] Mokhtarinia HR, Abazarpour S, Gabel CP (2020). Validity and reliability of the Persian version of the quick exposure check (QEC) in Iranian construction workers. Work.

[31] Mokhtarinia HR (2022). Cross-cultural adaptation, reliability, and validity of the work role functioning questionnaire 2.0 to Persian. Disabil Rehabil.

[32] Mokkink LB et al. COSMIN Study Design checklist for Patient-reported outcome measurement instruments. Amsterdam, The Netherlands, 2019: p. 1–32.

[33] Polit DF, Yang FM. Measurement and the measurement of change: a primer for the health professions. Volume 3. Wolters Kluwer Philadelphia; 2016.

[34] Anthoine E (2014). Sample size used to validate a scale: a review of publications on newly-developed patient reported outcomes measures. Health Qual Life Outcomes.

[35] Pugh JD (2015). Validity and reliability of an online extended version of the nordic Musculoskeletal Questionnaire (NMQ-E2) to measure nurses’ fitness. J Clin Nurs.

[36] Hernandez R (2022). Validation of the National Aeronautics and Space Administration Task load index (NASA-TLX) adapted for the whole day repeated measures context. Ergonomics.

[37] Grier RA (2015). How high is high? A Meta-analysis of NASA-TLX Global workload scores. Proc Hum Factors Ergon Soc Annual Meeting.

[38] Hendy KC, Hamilton KM, Landry LN. Hum Factors. 1993;35(4):579–601. [10.1177/001872089303500401]. Measuring Subjective Workload: When Is One Scale Better Than Many?.

[39] Hosseini ZSJ et al. Structured multidisciplinary work evaluation Tool (SMET) questionnaire: translation, cultural adaptation and psychometric evaluation of the Persian version. Work, 2023(Preprint): p. 1–11. 10.3233/WOR-220706 37742679

[40] Mohammadi M, Mazloumi A, Zeraati H (2013). Designing questionnaire of assessing mental workload and determine its validity and reliability among ICUs nurses in one of the TUMS’s hospitals. J School Public Health Inst Public Health Res.

[41] Beaton DE (2000). Guidelines for the process of cross-cultural adaptation of self-report measures. Spine.

[42] Fermanian J. Validation of assessment scales in physical medicine and rehabilitation: how are psychometric properties determined? in Annales de readaptation et de medecine physique: revue scientifique de la Societe francaise de reeducation fonctionnelle de readaptation et de medecine physique. 2005. 10.1016/j.annrmp.2005.04.004 15923054

[43] Rodrigues IB et al. Development and validation of a new tool to measure the facilitators, barriers and preferences to exercise in people with osteoporosis. BMC Musculoskelet Disord, 2017. 18(1): p. 540. [PMC5738121] [10.1186/s12891-017-1914-5]. 10.1186/s12891-017-1914-5 PMC5738121 29258503

[44] Polit DF, Beck CT, Owen SV (2007). Is the CVI an acceptable indicator of content validity? Appraisal and recommendations. Res Nurs Health.

[45] Terwee CB (2007). Quality criteria were proposed for measurement properties of health status questionnaires. J Clin Epidemiol.

[46] Munk R (2019). Measuring productivity costs in patients with musculoskeletal disorders: measurement properties of the Institute for Medical Technology Assessment Productivity cost questionnaire. Value in Health.

[47] Souza AC, Alexandre, Guirardello EdB (2017). Psychometric properties in instruments evaluation of reliability and validity. Epidemiologia E servicos de saude.

[48] Yona T (2020). The cross-cultural adaptation and reliability of the online hebrew version of the Extended Nordic Musculoskeletal Questionnaire. Musculoskelet Sci Pract.

[49] Fang YX (2013). [Test-retest reliability of Nordic Musculoskeletal Questionnaire in nurses]. Zhonghua Lao Dong Wei Sheng Zhi Ye Bing Za Zhi.

[50] Kahraman T, Genç A, Göz E. The Nordic Musculoskeletal Questionnaire: cross-cultural adaptation into Turkish assessing its psychometric properties. Disabil Rehabil, 2016. 38(21): p. 2153-60. [10.3109/09638288.2015.1114034]. 10.3109/09638288.2015.1114034 26726840

[51] Namnik N et al. Validity and reliability of Persian version of the Specific Nordic questionnaire in Iranian industrial workers. Work, 2016. 54(1): p. 35–41. [10.3233/wor-162268]. 10.3233/WOR-162268 26967033

[52] de Barros EN, Alexandre NM. Cross-cultural adaptation of the Nordic musculoskeletal questionnaire. Int Nurs Rev, 2003. 50(2): p. 101-8. [10.1046/j.1466-7657.2003.00188.x]. 10.1046/j.1466-7657.2003.00188.x 12752909

[53] Antonopoulou M et al. Translation and standardisation into Greek of the standardised general nordic questionnaire for the musculoskeletal symptoms. Eur J Gen Pract, 2004. 10(1): p. 33 – 4. [10.3109/13814780409094226]. 10.3109/13814780409094226 15060481

